# Osteopontin Is Induced by TGF-β2 and Regulates Metabolic Cell Activity in Cultured Human Optic Nerve Head Astrocytes

**DOI:** 10.1371/journal.pone.0092762

**Published:** 2014-04-09

**Authors:** Carolin Neumann, Fabian Garreis, Friedrich Paulsen, Christian M. Hammer, Marco T. Birke, Michael Scholz

**Affiliations:** 1 Department of Anatomy II, Friedrich-Alexander-University Erlangen-Nürnberg, Erlangen, Germany; 2 Department of Ophthalmology, Tufts University, Boston, Massachusetts, United States of America; University of Alabama, Birmingham, United States of America

## Abstract

The aqueous humor (AH) component transforming growth factor (TGF)-β2 is strongly correlated to primary open-angle glaucoma (POAG), and was shown to up-regulate glaucoma-associated extracellular matrix (ECM) components, members of the ECM degradation system and heat shock proteins (HSP) in primary ocular cells. Here we present osteopontin (OPN) as a new TGF-β2 responsive factor in cultured human optic nerve head (ONH) astrocytes. Activation was initially demonstrated by Oligo GEArray microarray and confirmed by semiquantitative (*sq*) RT-PCR, realtime RT-PCR and western blot. Expressions of most prevalent OPN receptors CD44 and integrin receptor subunits αV, α4, α 5, α6, α9, β1, β3 and β5 by ONH astrocytes were shown by *sq*RT-PCR and immunofluorescence labeling. TGF-β2 treatment did not affect their expression levels. OPN did not regulate gene expression of described TGF-β2 targets shown by *sq*RT-PCR. In MTS-assays, OPN had a time- and dose-dependent stimulating effect on the metabolic activity of ONH astrocytes, whereas TGF-β2 significantly reduced metabolism. OPN signaling via CD44 mediated a repressive outcome on metabolic activity, whereas signaling via integrin receptors resulted in a pro-metabolic effect. In summary, our findings characterize OPN as a TGF-β2 responsive factor that is not involved in TGF-β2 mediated ECM and HSP modulation, but affects the metabolic activity of astrocytes. A potential involvement in a protective response to TGF-β2 triggered damage is indicated, but requires further investigation.

## Introduction

Glaucoma is a generic term for a heterogeneous group of ocular neuropathies generally defined as progressive degeneration of retinal ganglion cells (RGCs) and loss of optic nerve axons. Without therapeutic intervention, this will lead to a confined visual field and finally to complete blindness. In the year 2020 it is estimated that more than 80 million people will suffer from a glaucomatous disease worldwide [1]. The molecular pathophysiology of glaucoma is poorly understood, reflecting its complex multifactorial etiology [2]. In regard to their etiology, glaucomas can be sub grouped into primary and secondary glaucomas. Whereas secondary glaucomas are caused by a distinct initial event, like trauma, steroid therapy, intraocular tumors or inflammatory processes such as uveitis, primary glaucomas develop in an idiopathic manner. The most common and best studied primary glaucoma worldwide is primary open-angle glaucoma (POAG). Today, an estimated 4.5 million people are blind due to a POAG, which represents more than twelve percent of all global causes for blindness [1]. Evident causes and the basic pathomechanisms of POAG are still not satisfyingly elucidated. Advanced age and elevated intraocular pressure (IOP) are the most important risk factors for developing a POAG. Clinical studies, various data from experimental animal models and morphology studies point out that the optic nerve head (ONH) and the lamina cribrosa are the initial sites of neurodegenerative processes [3]. The typical loss of axons is frequently accompanied by an accumulation of ECM in an unstructured distribution throughout the ONH known as ONH tissue remodeling [4]–[6]. It is generally accepted that the main source of the ECM within the ONH are astrocytes. We already demonstrated that cultured human ONH astrocytes respond with a strong increase of ECM protein secretion and produce high levels of the inhibitor of ECM degradation, PAI-1, when exposed to TGF-β2 [7]–[11], the most frequently increased aqueous humor (AH) factor in POAG patients [12], [13].

In a previous study we introduced osteopontin (OPN) as a novel AH factor that increases with age in DBA/2J mice, a widely used animal model for glaucomatous neurodegeneration in the eye [14]–[19]. Moreover, OPN showed a significant correlation with the progressive degree of optic nerve degeneration and RGC loss in these mice [20]. OPN is a secreted ECM protein with a broad variety of biological activities. It is encoded by the gene *secreted phosphoprotein* (*spp1*) and expressed by a wide spectrum of different cells during embryogenesis, wound healing, inflammation and tumorigenesis [21]. Under physiological conditions OPN expression is low, but slightly raised during inflammation [22], [23]. OPN protein levels are significantly increased in neurodegenerative diseases such as Alzheimer’s, Parkinson’s, multiple sclerosis and stroke [24]–[30]. The precise function of OPN in these conditions is not yet confirmed, but data suggest either a role as an active mediator of the degeneration process or as part of the neuroprotective response.

The present study was carried out to investigate the regulatory effects of TGF-β2 on OPN expression in cultured human ONH astrocytes. We also examined the expression of the most prevalent OPN receptors in cultivated ONH astrocytes and their responsiveness to TGF-β2. Furthermore, we analyzed potential regulatory effects of OPN regarding expression of (i) ECM proteins, (ii) proteins of the ECM degradation system, (iii) POAG-associated stress proteins, and (iv) the metabolic activity of cultured ONH astrocytes.

## Materials and Methods

### Ethics Statement

Methods included proper consent and approval, complied with the declaration of Helsinki, and were approved by the ethic committee of the Ludwig Maximilian University, Munich, Germany as described before [31].

### Human Optic Nerve Head (ONH) Astrocyte Explant Cultures

Human donor eyes from 11 donors with no history of eye diseases (19–62 years old, 4–8 h post mortem) were obtained from the eye bank and the Department of Ophthalmology of the Ludwig Maximilian University, Munich, Germany. Preparation of ONH astrocytes and their characterization was done as described before [8]. Monolayer ONH astrocytes were cultured in DMEM/F12 (1∶1; PAA Laboratories) supplemented with 10% FCS (Invitrogen) in a humidified 5% CO2 incubator at 37°C. For cell culture experiments ONH astrocytes of passages 3–5 were used.

### Cell Culture

Astrocytes were collected from subcultures and 2×10^3^ cells/well were seeded to 96 well plates (MTS assay) or 1×10^4^ cells/well in 6 well plates in DMEM/F-12 with 10% FCS. At confluence, before treatment cells were starved for 24 h in serum-free DMEM/F-12. Then medium was changed to serum-free DMEM/F-12 containing one or more of the fowling substances: 1 ng/ml active TGF-β2 (R&D Systems, 302-B2-010/CF), 250, 1000 or 2000 ng/ml human recombinant OPN (R&D Systems, 1433-OP-050/CF), 100 nM RGD peptide (Sigma, A8052), or an anti-CD44 blocking antibody (1∶100, Abcam, ab41478). In each experiment, control cultures were incubated with the solvent in serum-free medium alone.

### RNA Preparation and Complementary DNA (cDNA) Synthesis

Total RNA from cultured ONH astrocytes was extracted using TRIZOL reagent (Invitrogen). Crude RNA was purified with isopropanol and repeated ethanol precipitation, and contaminated DNA was destroyed by digestion with RNase-free DNase I (Boehringer). Structural integrity, yield and purity of RNA were determined photometrically and confirmed by electrophoresis. First-strand complementary DNA (cDNA) was amplified from 2.5 μg total RNA using a Superscript II reverse transcriptase kit (Invitrogen) according to the manufacturer’s protocol.

### Oligo GEArray Analysis

For the Oligo GEArray human extracellular matrix and adhesion molecules microarray (Sabiosciences OHS-013) astrocyte total RNA from one donor cell line (donor age: 54 years) was isolated and purified using an ArrayGrade total RNA isolation kit (SuperArray). The Oligo GEArray microarray was performed according to the manufacturer’s protocol (http://saweb2.sabiosciences.com/gene_array_product/HTML/OHS-013.html). Different gene expression from untreated and 1 ng/ml TGF-β2 treated ONH astrocytes were detected by chemiluminescence signals with a Lumi imager (Boehringer). Quantification was performed with the Lumi-Analyst software (Boehringer).

### *Semiquantitative* (*sq*) RT-PCR

For gene-specific RT-PCRs, each reaction was prepared with 5 μl cDNA, 2.5 μl 10× PCR buffer (Mg^2+^-free), 0.75 μl 50 mM MgCl_2_, 0.5 μl 10 mM dNTPs, 0.5 μl 10 μM primer mix, 0.1 μl Taq polymerase (5 U/μl; all solution from Invitrogen) in a total volume of 25 μl. PCR cycles were 30 s denaturation at 96°C, 30 s annealing and 45 s extension at 72°C, followed by a final extension for 5 min at 72°C. Primer sequences, annealing temperatures, cycle numbers and product sizes are given in table S1. The RT-PCR conditions and cycle numbers were chosen so that none of the gene-specific amplicons reached a plateau at the end of the PCR protocol, i.e. they were in the exponential phase of amplification. Functionality of primers was tested on cDNAs obtained from different tissues prior to the experiments to exclude false-negative results. Ten microliters of the PCR were loaded on a 1.5% agarose gel and after electrophoresis, PCR products were visualized by ethidium bromide staining. Fluorescence signals were detected with a Lumi imager (Boehringer) and quantification was performed with the Lumi-Analyst software (Boehringer). Band intensities were expressed as relative absorbance units. The ratio between the gene-specific PCR amplification product and reference gene *glyceraldehyde 3-phosphate dehydrogenase* (GAPDH) was calculated to normalize for initial variations in sample concentration and as a control for reaction efficiency. Mean and standard deviation (SD) of all experiments were calculated after normalization to GAPDH.

### Realtime (rt) RT-PCR

Induction of OPN gene expression was analyzed by real time RT-PCR using a LightCyler480 (Roche). Each reaction contained 5 μl cDNA, 4 μl LightCycler480 5× probe mastermix, 0.2 μl OPN forward primer (5′-gagggcttggttgtcagc-3′), 0.2 μl OPN reverse primer (5′-caattctcatggtagtgagttttcc-3′), 0.2 μl Universal ProbeLibrary (UPL) probe #18 (10 μM), and 12.5 μl nuclease-free water. OPN primers and the corresponding probe was performed using the ProbeFinder software (Version 2.04, Roche). Each plate was run at 95°C for 2 min, then 50 cycles of 95°C for 15 s, 60°C for 30 s, and 72°C for 30 s. A standard curve was generated by six-fold serial dilutions of cDNA from non-stimulated cells to examine PCR efficiency. To standardize mRNA concentration transcript levels of small ribosomal subunit (18S rRNA) were determined in parallel for each sample, and relative transcript levels were corrected by normalization based on the 18S rRNA transcript levels. All real-time RT-PCRs were performed in triplicate, and the changes in gene expression were calculated using the delta delta Ct method [32].

### Protein Extraction and Western Blot Analysis

ONH astrocytes were directly lysed in 250 μl RIPA lysis buffer (150 mM NaCl, 1% NP-40, 0.5% DOC, 0.1% SDS, 50 mM Tris pH 8) and protein purification was carried out as previously described [8]. 25 μl aliquots were separated by SDS-polyacrylamide gel electrophoresis (PAGE) and transferred onto a nitrocellulose membrane (Protran BA83, 0.2 μm; Schleicher & Schüll) at 70 V for 0.75 h in 1× transfer buffer (10 mM CAPS pH 11, 20% methanol, 0.1% SDS) by the tank blot method. Membranes were blocked in TBST/5% BSA (tris-buffered saline, 0.1% tween-20, 5% bovine serum albumin, pH 7.2) for 1 hour. After washing in TBST, the anti-osteopontin antibody (1∶500, Abcam, ab8448) was added in TBST/1% BSA for 1 hour at room temperature. After washing twice for 5 min with TBST, a horseradish peroxidase (HRP) conjugated secondary antibody (1∶10,000, Caltag) diluted in TBST/1% BSA was added for 30 min at room temperature. Blots were washed three times in TBST for 5 min and once in detection buffer. For detection CDP-star (Roche) was added to the membranes and chemiluminescence signals were visualized by exposure to light-sensitive films (Hyperfilm ECL; Amersham Biosciences/GE Healthcare) for 1–10 min. Quantification was done with the Lumi-Analyst software (Boehringer).

### Immunofluorescence (IF)

Cultured ONH astrocytes were grown on 4 well microscope chamber slides (Nunc). At semi-confluence cells were washed three times with PBS, fixed in methanol for 4 min and air-dried. Tissues from the human optic nerve head were embedded with OCT and 5 μm cryosections were done with a cryostat. Sections were thawed at room temperature and dried for 5 min. Labeling of OPN receptors in cultured human ONH astrocytes was done with primary antibodies against CD44 (St. Cruz, 1∶200), IntαV (St. Cruz, 1∶200), Intβ3 (St. Cruz, 1∶200) and Intβ5 (St. Cruz, 1∶200) and detection with an Alexa Fluor 488-conjugated secondary antibody (Mobitec, 1∶500–1∶2.000) as previously described [8]. Controls were incubated with non-immune IgGs and secondary antibodies alone to determine unspecific binding. Nuclei were counterstained with 4′,6-diamidino-2-phenylindole (DAPI) for 3 min and slides mounted with fluorescent mounting medium (Dako). Slides were analyzed under a Leitz Aristoplan fluorescence microscope.

### Metabolic Cell Activity Assay (MTS Assay)

The metabolic activity was assessed by the CellTiter 96 AQueous MTS Assay System (Promega) as described before [20]. In brief, ONH astrocytes were cultured in serum-free medium supplemented with 1 ng/ml active TGF-β2 or human recombinant OPN (250, 1000 and 2000 ng/ml), respectively. To test the effect of blocking OPN receptors, either 100 mM of an RGD-pathway blocking peptide (aa sequence: GRGDS, Sigma, A8052) or a CD44-blocking antibody (1∶100, Abcam, ab41478 [33]) was added. Metabolic activity was measured photometrically at the indicated time points in a plate reader (MWG) at 490 nm. Control cells were cultured in serum-free medium and analyzed at the same time points.

### Statistical Analysis

All data are represented as the mean average (m.a.) ± standard deviation (SD). OligoArrays were performed on 1 human ONH astrocyte line derived from 1 donor (aged 54 years). For all other experiments (RT-PCR, rtPCR, WB, IF and MTS assay) 11 different human ONH astrocyte lines derived from 11 different donors (aged 19 to 62 years) were used. Statistical significance was evaluated by a student’s t-test using the InStat statistical software. P values of less than 0.05 were considered as statistically significant.

## Results

### TGF-β2 Activates Osteopontin (OPN)

**Figure 1:**
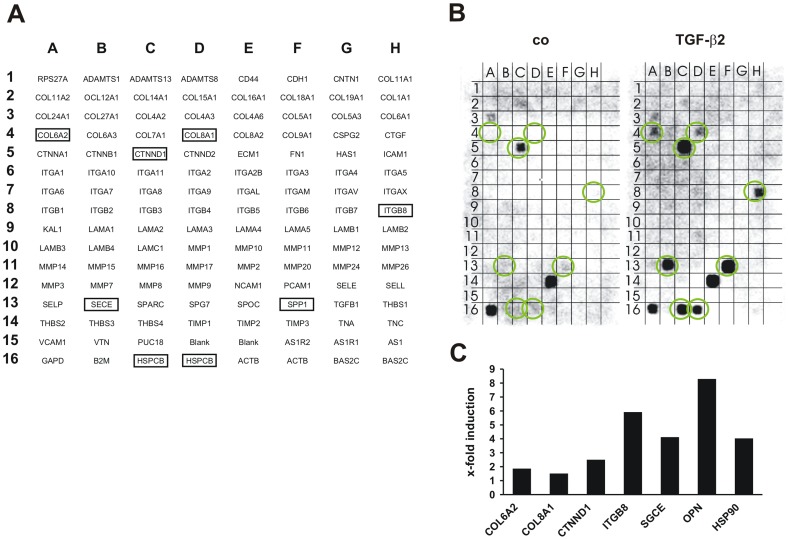
Results of the Oligo GEArray microarray. Densitometric measurements demonstrate induction of seven putative TGF-β2 responsive genes in human optic nerve head (ONH) astrocytes. (A) Oligo GEArray Human Extracellular Matrix and Adhesion Molecules microarray layout. (B) Chemiluminescence signals of microarrays incubated with total mRNA from ONH astrocytes treated with 1 ng/ml TGF-β2 and control cells (co). Regulated genes are marked. (C) Densitometric analysis of the gene induction of TGF-β2 responsive genes in ONH astrocytes compared to untreated cells. TGF-β2-dependent up-regulation of collagen 6α2 (COL6A2), collagen 8α1 (COL8A1), catenin delta 1 (CTNND1), integrin beta 8 (ITGB8), epsilon sarcoglycan (SGGE), osteopontin (OPN) and heat shock protein 90 (HSP90).

TGF-β2 is one of best characterized aqueous humor factors in the context of PAOG and was shown to impact protein expression in ONH astrocytes. In an initial experiment we screened for novel TGF-β2 responsive factors in cultured human optic nerve head (ONH) astrocytes. We performed an Oligo GEArray microarray for 113 genes involved in cell adhesion and remodeling of extracellular matrix (Figure 1A). Seven putative TGF-β2 responsive genes were identified in ONH astrocytes treated with 1 ng/ml TGF-β2 compared with untreated cells (Figure 1B). Densitometric measurements of the signal intensity of the spotted genes revealed increases in collagen 6α2 (COL6a2, 1.8-fold), collagen 8α1 (COL8α1, 1.5-fold), catenin delta 1 (CTNND1, 2.5-fold), integrin beta 8 (ITGB8, 5.9-fold), epsilon sarcoglycan (SGCE, SECE, 4.1-fold), heat shock protein 90 (HSP90, HSPCB, 4.0-fold) and osteopontin (OPN, *secreted phosphoprotein 1* SPP1). As the OPN gene signal displayed the highest increase by about 8.3-fold compared to untreated ONH astrocytes (Figure 1C).

**Figure 2:**
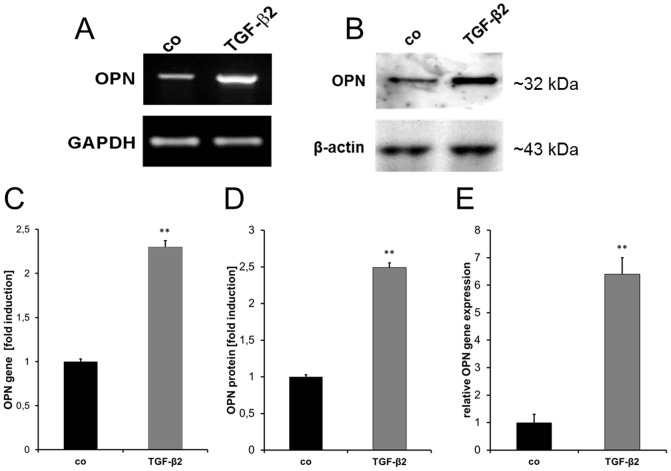
Quantification of OPN induction in TGF-β2 treated ONH astrocytes. Analysis of OPN expression in ONH astrocytes after treatment with 1/ml TGF-β2 for 72 hours. (A) S*emiquantitative (sq)* RT-PCR analysis indicates an up-regulation of OPN transcript in TGF-β2 treated ONH astrocytes compared to control cells (co). (B) Western blot analysis demonstrates an increase in secreted OPN protein (MMP-cleaved) in TGF-β2 treated ONH astrocytes compared to controls. (C) Densitometric quantification of *sq* RT-PCR reveals significant induction of OPN mRNA (2.3-fold, p = 0.008). OPN signals are normalized to GAPDH. (D) Quantification of western blot results reveals significantly increased OPN secretion into supernatant (2.5-fold, p = 0.0054). OPN western blots are normalized within the β-actin signal on the same nitrocellulose membrane. (E) Statistical analysis of real time RT-PCR shows significant up-regulation of OPN transcript (6-fold, p = 0.0073). Values represent mean ± SD of 11 independent experiments (n = 11).

In further investigation, induction of the putative candidate genes was analyzed by *semiquantitative* (*sq*) RT-PCR experiments to verify the microarray data. Densitometric quantifications of the *sq* RT-PCR data did not confirm up-regulation for COL8α1, CTNND1, ITGB8, SGCE and HSP90 gene expression (Figure S1). Induction of the OPN gene was confirmed in contrast to this and quantified as 2.3-fold (p = 0.008) compared to untreated ONH astrocytes (Figure 2A, C). In additional realtime RT-PCR experiments, we detected a 6.0-fold up-regulation (p = 0.0073) upon treatment with 1 ng/ml active TGF-β2 (Figure 2E). Western blot experiments revealed a 2.5-fold increase of the secreted OPN protein (MMP-cleaved) in the culture medium (p = 0.0054, Figure 2B, D) compared to the corresponding controls.

### ONH Astrocytes Express OPN Receptors

**Figure 3:**
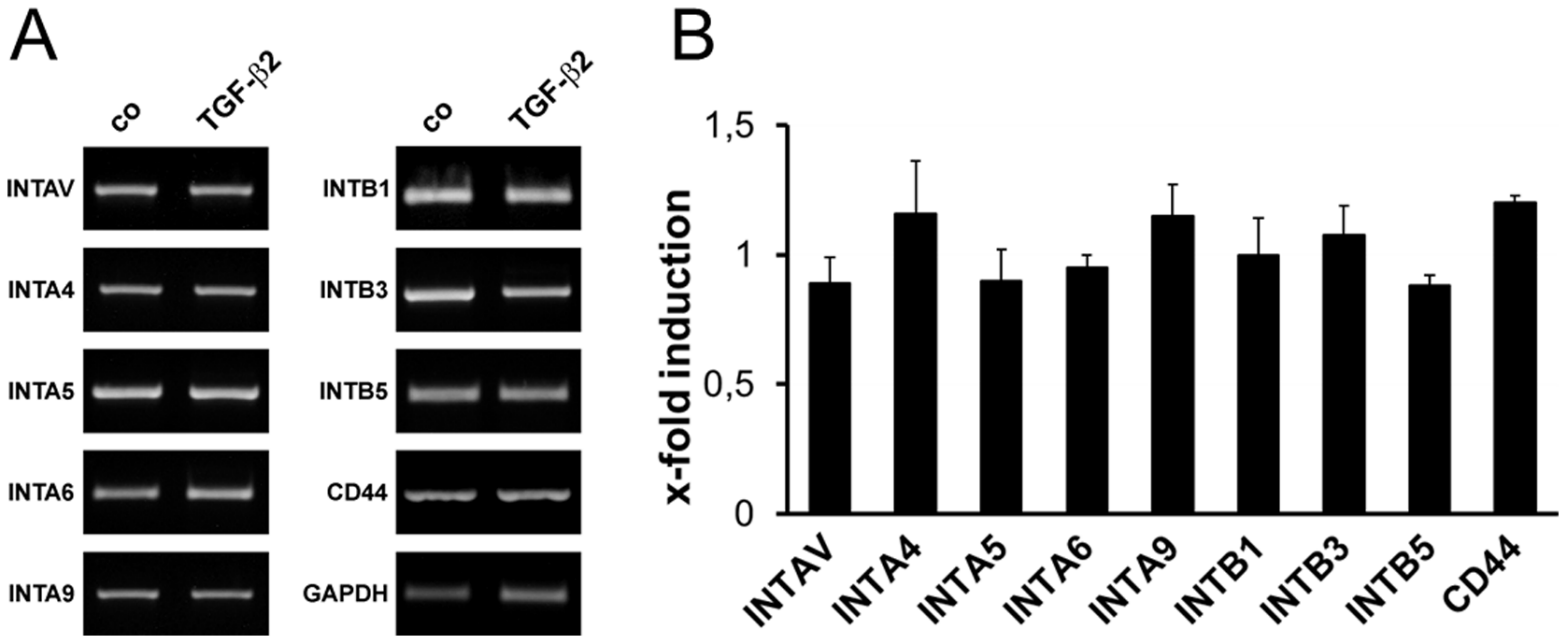
*Semiquantitative* RT-PCR analyses of most prevalent OPN receptors in cultivated ONH astrocytes and effect of TGF-β2. (A) Representative RT-PCR results of OPN receptor gene expression in untreated (co) and TGF-β2 (1 ng/ml, 72 h) treated ONH astrocytes. (B) Densitometric analysis of *sq* RT-PCR results does not demonstrate any regulation of OPN receptors in TGF-β2 treated cells compared to controls. OPN signal is normalized to GAPDH. Values represent mean ± SD of 11 independent experiments (n = 11).

Expression of the most prevalent OPN receptor CD44 and integrin-type OPN receptor subunits in cultivated human ONH astrocytes was analyzed by RT-PCR and immunofluorescence (IF). RT-PCR analysis confirmed gene expression of CD44 and the most prevalent integrin OPN receptor subunits IntαV, Intβ3 and Intβ5. Moreover, gene expression of the integrin subunits Intα4, Intα5, Intα6, Intα9 and Intβ1 was also demonstrated (Figure 3A). Densitometric analysis of *sq* RT-PCR results revealed that TGF-β2 (1 ng/ml) had no regulatory effect on any of the tested receptors or integrin receptor subunits (Figure 3B). GAPDH gene expression served as control for equal cDNA amounts and was considered for quantification.

**Figure 4:**
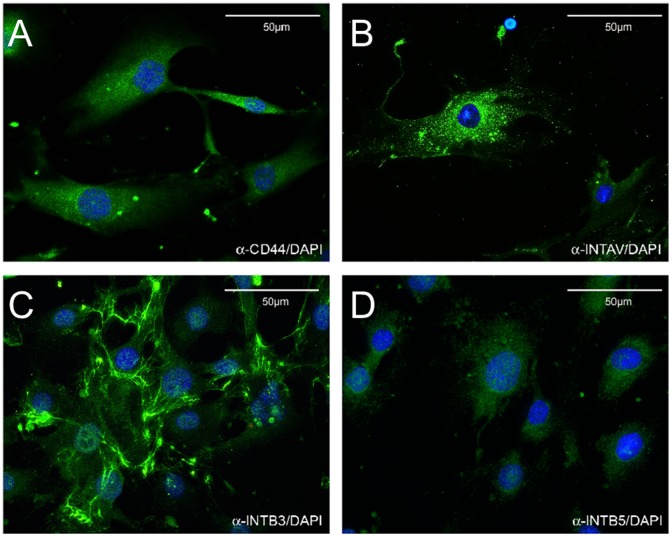
Localization of most prevalent OPN receptors on cultured human ONH astrocytes. Immunofluorescence signals (green) indicate expression of CD44 (A) and integrin receptor subunits IntαV (B), Intβ3 (C) and Intβ5 (D). The nuclei (blue) are counterstained with DAPI. Pictures are representative for three independent experiments. Scale bar 50 μm.

To confirm expression of proteins and to assess the cellular localization of CD44 and the receptor subunits IntαV, Intβ3 and Intβ5 immunofluorescence labelings of cultivated ONH astrocytes were performed. Signals for CD44 and the integrin subunits were restricted to the surface of the outer cell membrane. Antibody reactivity for CD44 and Intβ5 was evenly distributed over the entire cell surface, whereas the localization of IntαV was distributed in a speckled pattern. Localization of Intβ3 was also distributed over the entire cell surface, but very intense reactivity appeared to follow the cytoskeleton of the ONH astrocytes (Figure 4A–D).

### OPN does not Affect Expression of Glaucoma-associated ECM Components

**Figure 5:**
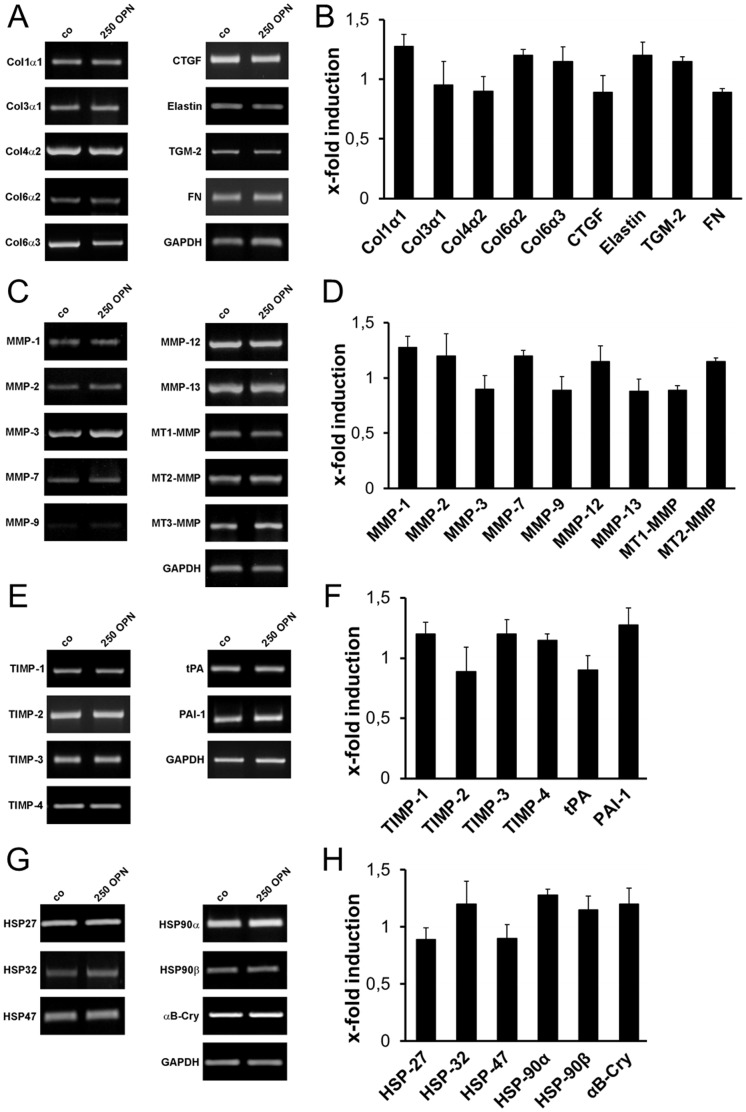
*Semiquantitative* RT-PCR analysis of glaucoma-associated ECM components in ONH astrocytes after treatment with OPN. Treatment with 250/ml OPN for 72 hours has no effect on gene expression of glaucoma-associated ECM components (A, B), genes of the ECM degradation system (C–F), as well as stress response genes (G, H) in cultivated ONH astrocytes compared to controls (0 OPN). (A, C, E and G) Representative RT-PCR results upon treatment with 250 ng/ml OPN () and controls (0 OPN). (B, D, F and H) Densitometric analysis of *sq* RT-PCR results normalized to GAPDH. Values represent mean ± SD of 11 independent experiments (n = 11).

**Figure 6:**
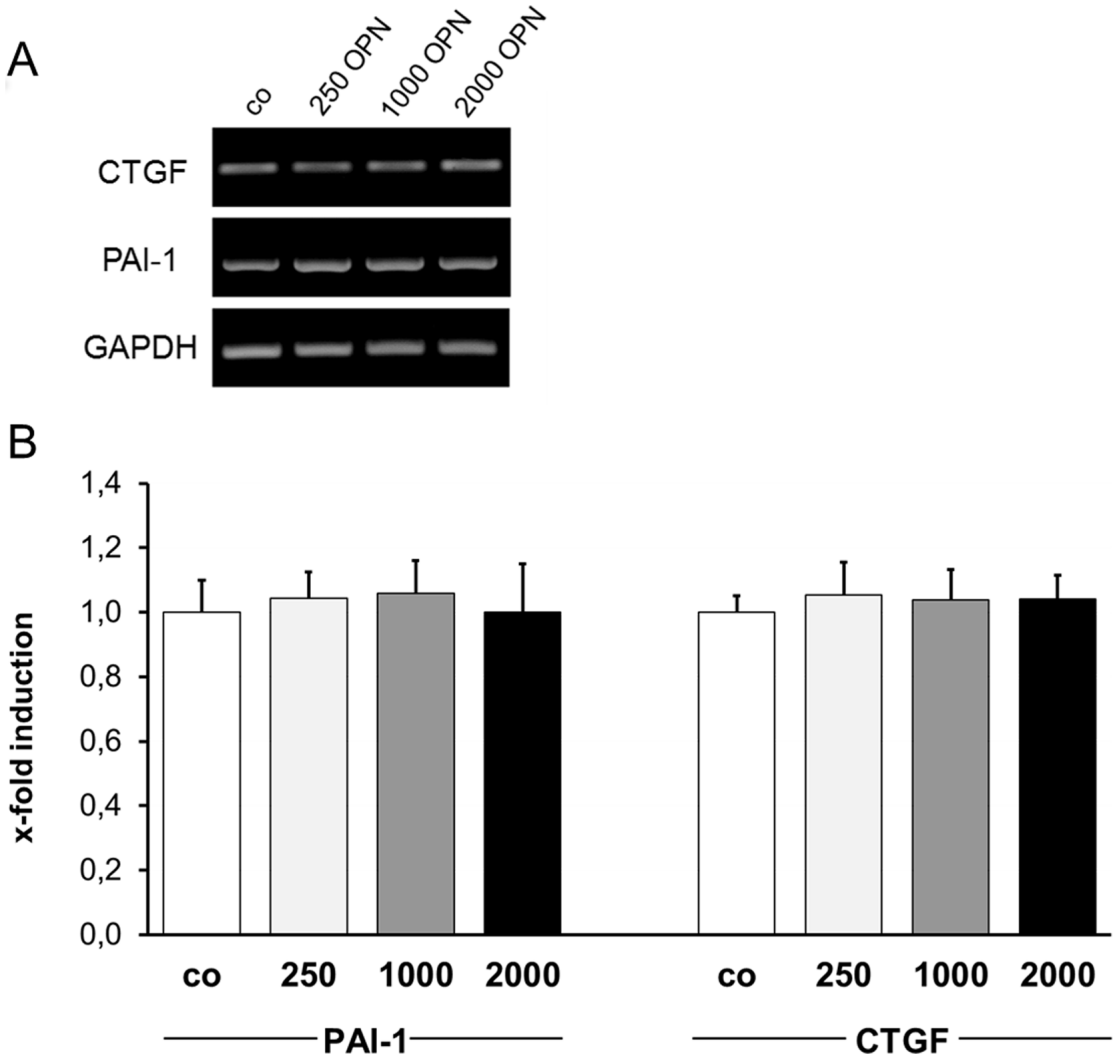
*Semiquantitative* RT-PCR analysis of key regulator of ECM synthesis in ONH astrocytes after treatment with different OPN concentrations. Increasing OPN concentration has no effects to the gene expression of key regulator of ECM synthesis (CTGF) or degeneration (PAI-1) in cultivated ONH astrocytes. (A) Representative RT-PCR results of connective tissue growth factor (CTGF) and plasminogen activator inhibitor-1 (PAI-1) in untreated (co) and OPN treated ONH astrocytes. (B) Densitometric analysis of *sq* RT-PCR results reveal no gene induction of CTGF and PAI-1 after treatment with 250, 1000 or 2000 ng/ml OPN compared to untreated cells (co). OPN signal is normalized to GAPDH. Values represent mean ± SD of 11 independent experiments (n = 11).

Effects of OPN on gene expression of glaucoma-associated ECM components, ECM-degrading enzymes, their inhibitors and heat shock proteins were analyzed by *sq* RT-PCR. ONH astrocytes were cultivated in the presence of 250 ng/ml OPN for 72 hours and subjected to *sq* RT-PCR analysis. At this concentration, OPN did not alter the expression level of any of the tested ECM components: collagen type 1α1, -3α1, -4α2, -6α2 and -6α3, elastin, connective tissue growth factor (CTGF), transglutaminase 2 (TGM-2) and fibronectin (FN) (Figure 5A, B). The same result was observed for matrix metalloproteinases (MMPs)-1, -2, -3, -7, -9, -12, -13, membrane-type (MT)-MMPs-1, -2, -3, tissue inhibitors of metalloproteinase (TIMP)-1, -2, -3, -4, tissue plasminogen activator (tPA) and plasminogen activator inhibitor (PAI)-1 (Figure 5C - F). Expressions of heat shock proteins (HSP)-27, -32, -47, -90 and αB-crystallin (αB-Cry) also remained unaltered upon treatment with 250 ng/ml OPN (Figure 5G, H). In further experiments we analyzed the effects of higher OPN concentrations (1,000 and 2,000 ng/ml) on key regulators of ECM modification, CTGF and PAI-1. Densitometric quantification of *sq* RT-PCR results revealed no influence of higher OPN concentration on gene expression of CTGF and PAI-1 compared to untreated ONH astrocytes (Figure 6A, B). In all analyses, the gene expression of the reference gene GAPDH served as a control for equal cDNA amounts and was considered for quantification of regulatory effects.

### OPN Affects ONH Astrocyte Metabolism

**Figure 7:**
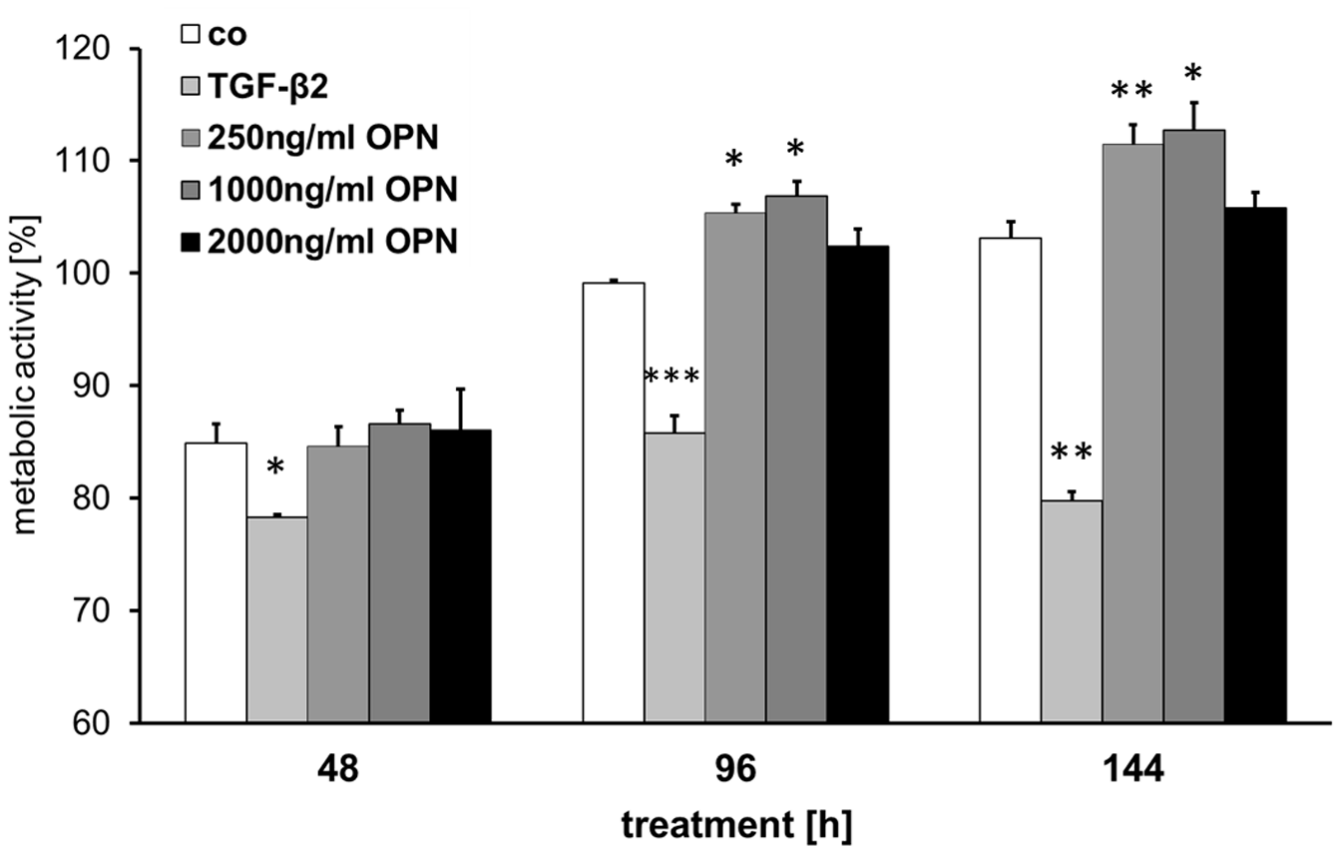
TGF-β2 and OPN effects on metabolic activity in cultured ONH astrocytes. Cell viability was assessed by MTS assays. Quantification of the metabolic cell activity of ONH astrocytes treated with TGF-β2 (1 ng/ml) and different OPN concentrations (0, 250, 1000, 2000 ng/ml) for 48, 96 and 144 hours. Values represent mean ± SD of 11 independent experiments (n = 11). Statistical significance was calculated by student’s t-test (*p<0.05; **p<0.01; ***p<0.001).

As we showed OPN signaling is possible in astrocytes, we wished to test potential effects of OPN on astrocytes in respect of cell activation or repression. Therefore we choose the MTS assay, which measures the mitochondrial activity of cells. Metabolic activity of human ONH astrocytes was measured after 48, 96 and 148 hours incubation with either 1 ng/ml TGF-β2 or 250, 1000 and 2000 ng/mL OPN. Values are presented as percent of starting activity (t = 0 h), which was set to 100% (Figure 7). At 48 h, metabolic activities of OPN incubated astrocytes did not differ from the controls, independent of the OPN concentration. At 96 h, astrocytes grown in 250 ng/ml and 1000 ng/ml OPN had significantly higher activities than the controls (p_250_ = 0.0223, p_1000_ = 0.022), whereas the 2000 ng/ml group did not differ from the controls. Quantification at 144 h showed a similar result, i.e. activities of the 250 ng/ml and 1000 ng/ml astrocytes were significantly increased (p_250_ = 0.0056, p_1000_ = 0.0105) and activity of the 2000 ng/ml group was in the range of the controls. Astrocytes incubated with TGF-β2, in contrast, showed significantly lower activities at all-time points compared to control astrocytes (p_48_ = 0.0312, p_96_ = 0.0007, p_144_ = 0.0026) and all astrocytes grown in presence of OPN (p<0.05 for each approach).

### Selective Blocking of OPN Receptor Signaling

**Figure 8:**
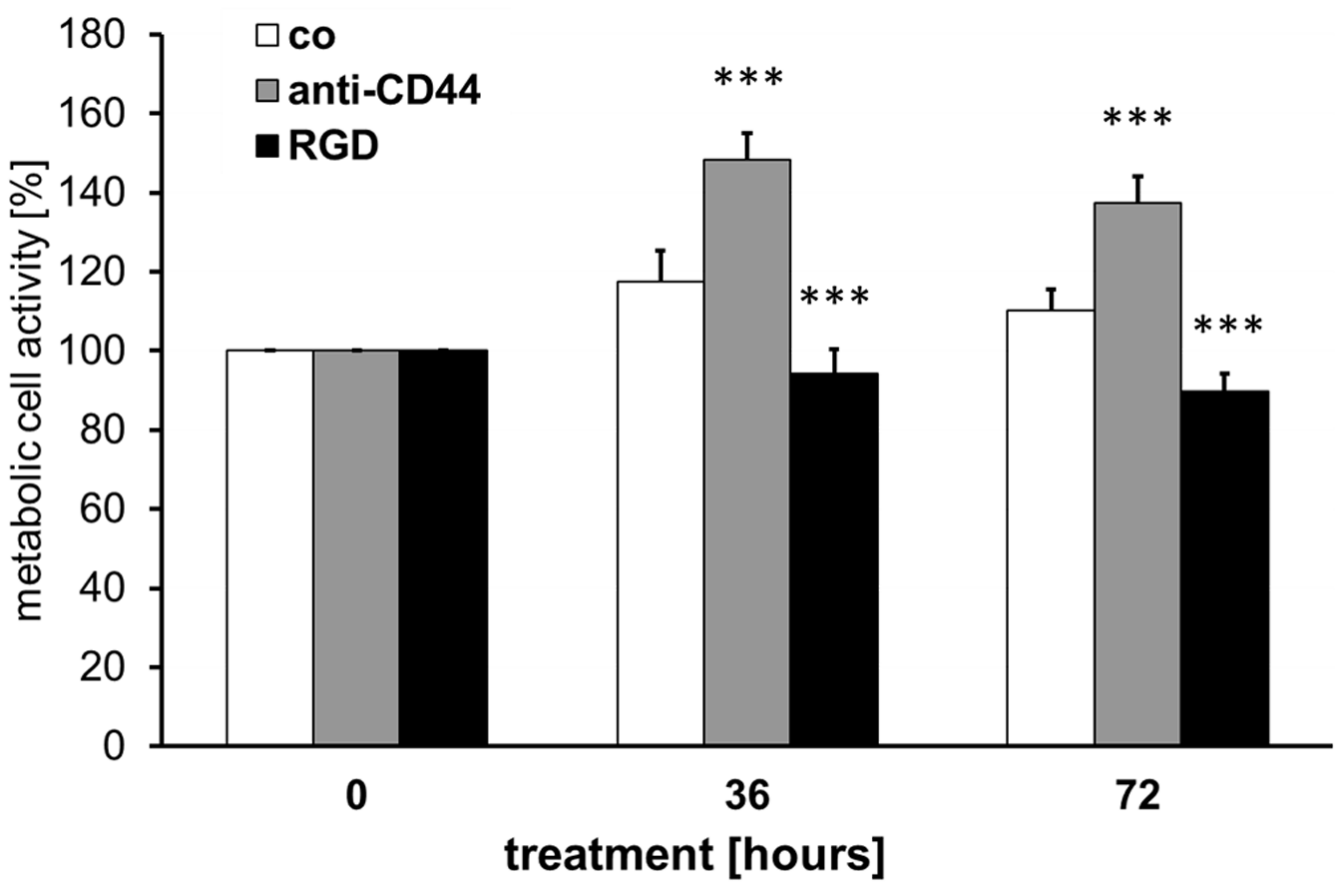
Effects of selective OPN receptor blocking on metabolic activity in cultured ONH astrocytes. Metabolic cell activity of ONH astrocytes incubated with an anti-CD44 antibody or a synthetic RGD peptide for 36 and 72 hours compared to untreated cells (co). Values represent mean ± SD of 11 independent experiments (n = 11). Statistical significance is calculated by student’s t-test (*p<0.05; **p<0.01; ***p<0.001).

To get an insight how signaling via the two different OPN receptor types expressed by astrocytes – CD44 and integrins – influence metabolic activity, we blocked either the integrin receptors by addition of a synthetic RGD peptide (aa-sequence: GRGDS) or CD44 with a CD44 blocking antibody (anti-CD44). Inhibition of OPN signal transmission through CD44 led to a significant increase of metabolic activity of cultured ONH astrocytes after 36 and 72 hours compared to control cells (p<0.001 each approach, Figure 8). Blocking of OPN signaling via integrin receptors had the opposite effect, in contrast, i.e. the metabolic activity was statistically significantly reduced compared to controls and anti-CD44 supplemented ONH astrocytes (p<0.001 each approach, Figure 8).

## Discussion

Our results clearly identify OPN as a novel TGF-β2 responsive factor in cultured human optic nerve head astrocytes. Commercial Oligo GEArray, realtime RT-PCR and western blot analysis exhibit a significant increase of OPN gene and protein expression in response to TGF-β2 treatment. Recent studies showed that TGF-β2 is associated with POAG with an increase of expression and synthesis of glaucoma-associated ECM components [8]–[10], [13], [34], [35]. It also has been shown previously that TGF-β2 and other growth factors, such as insulin-like growth factor 1 (IGF-1), epidermal growth factor (EGF) and TGF-β1 can induce up-regulation of OPN expression in various cell types [36]–[38]. However, this effect of TGF-β2 was not yet demonstrated in ONH astrocytes. In a previous study we introduced OPN as an age-related increased AH factor correlated with optic nerve degeneration and loss of retinal ganglion cells (RGCs) in the DBA/2J mouse model for glaucomatous and neurodegenerative changes in the eye [20]. Additionally, several previously published studies show that OPN correlates with various neurodegenerative pathologic conditions such as Alzheimer’s, Parkinson’s, multiple sclerosis and stroke [24]–[30]. In relation to other diseases, e.g. cardiovascular or pulmonary diseases, it is known that OPN expression is frequently up-regulated in response to mechanical and oxidative stress as well as injury and inflammation in a variety of different tissues [39]–[42]. In rodent models of neurodegenerative diseases, locally elevated levels of OPN have been identified in activated glia cells adjacent to the lesion site [27], [28], [43]. Chidlow et al. [44] showed OPN expression in RGCs under physiological conditions in the rat retina and that activated microglia synthesize OPN *de novo* in response to excitotoxic and ischemic injury. Glia cell line-derived neurotrophic factor (GDNF)-induced OPN from Müller cells and promoted photoreceptor survival in the Pde6b^rd1^ mouse model of retinal degeneration [45]. Moreover, it was recently reported that OPN also inhibits the swelling of rat Müller cells (i) induced by hypoosmotic exposure of retinal slices in the presence of both barium ions and H_2_O_2_,and (ii) in slices of postischemic retinas [46]. In summary, there are strong indications that OPN either mediates triggers, triggers or even induces neuroprotective responses in the CNS. In the context of POAG such a response could be induced to counteract degeneration from ischemia, subacute chronic inflammation and increased IOP to protect RGCs and optic nerve axons but maybe also glia and astrocytes.

In further investigations, we analyzed OPN signaling in ONH astrocytes focused on the expression of specific OPN receptors. It is known that OPN interacts with ubiquitously expressed cell surface receptors, including RGD-dependent integrin subunits and the RGD-independent CD44 receptor, to mediate cell adhesion, migration and survival in a variety of cell types [21], [47]. By RT-PCR analysis and immunofluorescence the most prevalent integrin receptor subunits IntαV, Intβ3, Intβ5 and non-integrin receptor CD44 were detected in cultivated ONH astrocytes. Furthermore, *sq* RT-PCR results do not show regulation of OPN receptors in cultivated ONH astrocytes after treatment with TGF-β2. These data suggest existence of a receptor/ligand interaction by which OPN acts on astrocytes and which is not sensitive to TGF-β2.

In previous studies, changes in ECM remodeling associated with TGF-β2 expression and POAG are observed within the trabecular meshwork and the lamina cribrosa of the optic nerve *in vitro* and *in vivo* [5]–[8], [11], [35], [48]–[50]. Here, we analyzed the effect of 250 ng/ml recombinant OPN, which corresponds to the OPN concentration in the aqueous humor in mice [20], on cultivated ONH astrocytes regarding glaucoma-associated ECM components as well as proteins of the ECM degradation system. Our *sq* RT-PCR results demonstrate no effect on the gene expression of ECM components associated with glaucoma. Also, higher OPN concentrations (1.000 and 2.000 ng/ml) do not influence gene expression of connective tissue growth factor (CTGF), which is up-regulated by TGF-β2 and is a strong inducer of ECM proteins in cultivated human astrocytes and trabecular meshwork cells [7], [8], [51], [52]. Moreover, accumulation of ECM components can also be the result of an altered expression of components of the ECM degradation system. Apart from a basal expression in cultivated ONH astrocytes, no up-regulation of matrix metalloproteinases (MMPs), tissue inhibitors of metalloproteinases (TIMPs) and genes involved in the activation of pro-MMP, e.g. tissue plasminogen activator (tPA) or plasminogen activator inhibitor (PAI)-1, is detectable at the mRNA level after OPN treatment. Gene expression of PAI-1, an inhibitor of the plasminogen activation system induced by TGF-β2 [53], is not influenced by higher OPN concentrations. Furthermore, our *sq* RT-PCR analysis with heat shock proteins (HSP), frequently described for glaucomatous diseases, revealed no activation of stress response in ONH astrocytes by OPN. Yu *et al.* were able to show in a previous study that H_2_O_2_-induced oxidative stress and TGF-β2 reactivate ONH astrocytes by increasing Hsp32 and -47 expression *in vitro* [54]. In summary, we conclude from these data that OPN is not involved in the TGF-β2 induced activation of POAG-associated ECM and stress response genes. However, other studies show that OPN is required for the activation, migration, proliferation, and differentiation of fibroblasts and is up-regulated in several fibrotic diseases [55]–[57]. In a dystrophic mouse model (*mdx* mice), OPN promotes fibrosis in muscle by modulating immune cell subsets and intramuscular TGF-beta [58]. Abu El-Asrar *et al.* [59] demonstrated that OPN and other regulators of angiogenesis and fibrogenesis contribute to the pathogenesis of proliferative vitreoretinal disorders in humans. Another study showed that OPN is expressed in injured lens epithelial cells in association with fibrotic scar formation in mice and humans [60].

In early stages of POAG, an increase of astrocytes corresponding to an astrogliosis is often detectable. In late-stage glaucoma, however, a decreased count of astrocytes in the optic nerve is observed [4], [6], [49], [50], [61]. These findings suggest that proliferation and/or cell survival are deregulated in glaucomatous eyes. For that reason we analyzed the effect of OPN and TGF-β2 on the cell viability of cultivated human ONH astrocytes by means of metabolic activity assays. Our results show a time- and dose-dependent pro-metabolic effect of OPN on cultured human ONH astrocytes. This positive effect on cell viability might also indicate a neuroprotective impact of OPN. Tambuyzer *et al.,* [62] showed that OPN containing medium of a pig renal epithelial cell line (LLC-PK1) doubled the rate of proliferation of porcine microglia *in vitro*. The additional application of an anti-OPN polyclonal antibody completely reversed this effect [62]. Interestingly, a higher OPN concentration (2.000 ng/ml) does not further increase the metabolic activity of ONH astrocytes. These data are in line with a previous study, which indicated a limiting OPN concentration and anti-proliferative effect at higher OPN doses in murine neuronal precursor cells (RGC5) and *ex vivo* cultivated DBA/2J eyes in a long-time cultivation assay [20]. In contrast, ONH astrocytes treated with TGF-β2 displayed significantly reduced metabolic activity at all observed time points. This result shows that ONH astrocytes are TGF-β2 sensitive and indicate an anti-proliferation effect on these cells. Thus, it is tempting to speculate that OPN might be involved in counteracting the anti-proliferation TGF-β2 effect on ONH astrocytes.

Even our OPN receptor blocking experiments in cultivated astrocytes provide pro- and anti-metabolic effects by blocking OPN specific receptors. Blocking of RGD-independent CD44 led to increased metabolic activity, thus perhaps indicating an activation of the anti-proliferative pathway via the CD44 receptor. This effect may be ONH astrocyte-specific. In IL-3-dependent bone marrow cells, OPN promotes proliferation and survival via CD44 [63]. Blocking of RGD-dependent integrin receptors otherwise significantly reduced metabolic activity in astrocytes, which could be a link to a pro-proliferation signal transduced by integrin receptors. Meller *et al.* [28] demonstrated that OPN is a potent neuroprotective factor against ischemic injury depending on integrin-binding RGD motif and the activation of Akt and p42/p44 MAPK pathways. Several other studies have indicated that OPN mediates a pro-survival and anti-apoptotic signal to different cell types by inhibiting apoptosis induced by different pathological events and lack of growth factors [21]. Our initial data, however, require supplementation by further studies focusing on the correlation of OPN and astrocyte counts in different clinical stages of POAG eyes. The above-mentioned astrogliosis and degeneration of astrocytes is very likely only an accompanying phenomenon of glaucoma, but the disease does correlate with the degeneration of optic nerve axons and retinal ganglion cells.

In conclusion, we show in this study that OPN is a novel TGF-β2 responsive factor in cultured human ONH astrocytes and might be part of a rescue mechanism to counteract neurodegenerative effects of glaucoma-associated TGF-β2. It is conceivable that OPN is secreted by ONH astrocytes to protect the neurons of the optic nerve from mechanical and oxidative stress. Nevertheless, further studies are required to confirm a neuroprotective function of OPN in relation to retinal ganglion cells (RGCs) and optic nerve axons in glaucoma models.

## Supporting Information

Figure S1 **RT-PCR confirmation of the Oligo GEArray microarray data.** (A*) Semiquantitative* (*sq*) RT-PCR analysis of COL8α1, CTNND1, ITGB8, SGCE, HSP90 and OPN gene expression in untreated (co) and TGF-β2 treated astrocytes. (B) Densitometric analysis of the *sq* RT-PCR results. Signals are normalized to GAPDH. Values represent mean ± SD of 11 independent experiments (n = 11). Statistical significance is calculated by student’s t-test (**p≤0.01).
(TIFF)

Table S1 **Primers and settings used for** ***semiquantitative*** **(** ***sq*** **) RT-PCR analysis.** A_t_ = annealing temperature.
(DOCX)
