# Supplementary material for: Osteopontin Is Induced by TGF-β2 and Regulates Metabolic Cell Activity in Cultured Human Optic Nerve Head Astrocytes
Source: PLoS One. 2014 Apr 9;9(4):e92762. doi: 10.1371/journal.pone.0092762 (PMC3981660; doi:10.1371/journal.pone.0092762)
Supplement: Table S1 — Primers and settings used for semiquantitative ( sq ) RT-PCR analysis. At = annealing temperature. (DOCX) [file pone.0092762.s002.docx]

| Gene | Forward primer | Reverse Primer | Gene position | A_t_ [°C] | PCR cycles | Size [bp] |
| --- | --- | --- | --- | --- | --- | --- |
| CD44 | 5’-accgaccttcccattcacag-3’ | 5’-cactacaccccaatcttcat-3’ | 681-1233 | 55 | 35 | 553 |
| Col Iα1 | 5’-gatggactcaacggtctcc-3’ | 5’ccttggggttcttgctgatg-3’ | 325-1067 | 54 | 32 | 742 |
| Col IIIα1 | 5’-gacctgaaattctgccatcc-3’ | 5’-caaccatcctccaggactgtg-3’ | 2996-3456 | 54 | 35 | 461 |
| Col IVα2 | 5’-aaccaggttttcgtggggct-3’ | 5’-ttccggctggcatagtagca-3’ | 4568-4970 | 61 | 30 | 403 |
| Col VIα3 | 5’-tttcgactcctccctggtgttc-3’ | 5’-acaaaaagtcaggatgcccg-3’ | 2043-2581 | 58 | 30 | 538 |
| CTGF | 5’-cacaagggcctcttctgtga -3’ | 5’-tctcttccaggtcagcttcg -3’ | 342-858 | 60 | 32 | 517 |
| Elastin | 5’-gctttggcccgggagtagtt-3’ | 5’-caccttggcagcggattttg-3’ | 983-1602 | 60 | 30 | 619 |
| FN | 5’-gaagctctctctcagacaacca-3’ | 5’-aggtctgcggcagttgtcac-3’ | 6514-7182 | 56 | 27 | 669 |
| GAPDH | 5’-gaaggtgaaggtcggagtc-3’ | 5’-gaagatggtgatgggatttc-3’ | 108-333 | 57 | 23 | 225 |
| HSP 27 | 5’-gtccctggatgtcaaccact-3’ | 5’-ctttacttggcggcagtctc-3’ | 446-775 | 58 | 22 | 330 |
| HSP 32 | 5’-acatctatgtggccctggag-3’ | 5’-gtgtaaggacccatcggaga-3’ | 247-900 | 58 | 30 | 654 |
| HSP 47 | 5’-ttgttcggaggaagtcccctgtt-3’ | 5’-cgctcagcactgccttgg-3’ | 129-396 | 60 | 40 | 267 |
| HSP90α | 5’-tggtcctgtgcggtcact-3’ | 5’-tgcagctctttcccagagtct-3’ | 165-400 | 58 | 25 | 256 |
| HSP90β | 5’-gtctgggtatcggaaagcaag-3’ | 5’-tgagggttggggatgatgtc-3’ | 22-304 | 50 | 25 | 303 |
| INTA4 | 5’-ggatgtccagactactactgg-3’ | 5’-gaagccagccttccacataac-3’ | 3292-3775 | 65 | 40 | 484 |
| INTA5 | 5’-catttccgagtctgggccaa-3’ | 5’-tggaggcttgagctgagctt-3’ | 2877-3200 | 65 | 30 | 324 |
| INTA6 | 5’-ggcgagcaagctatgaaatc-3’ | 5’-gcagcagcagtcacatcaat-3’ | 2649- 3157 | 59 | 27 | 509 |
| INTA9 | 5’-tgagcatgtgactggagagg-3’ | 5’-cccagacaggtggcttgtat-3’ | 1799-2249 | 60 | 30 | 451 |
| INTAV | 5’-gagcagcaaggactttggg-3’ | 5’-gggtacacttcaagaccagc-3’ | 1090-1708 | 58 | 35 | 619 |
| INTB1 | 5’-tgttcagtgcagagccttca-3’ | 5’-cctcatacttcggattgacc-3’ | 2159-2610 | 53 | 30 | 452 |
| INTB3 | 5’-gaggatgactgtgtcgtcag-3’ | 5’-ctggcgcgttcttcctcaaa-3’ | 2076-2305 | 60 | 30 | 230 |
| INTB5 | 5’-caggatggggagaaccagagc-3’ | 5’-ctggtcatctttcacgatggt-3’ | 1798-2328 | 57 | 37 | 531 |
| INTB8 | 5’-ttcatcattttcatagttacattc-3’ | 5’-cattaagtgtttaaaaatcttttt-3’ | 2764-3039 | 54 | 35 | 276 |
| MMP-1 | 5’-gccagatttgccaagagcaga-3’ | 5’-cggcaaattcgtaagcagcttc-3’ | 437-1077 | 55 | 29 | 620 |
| MMP-2 | 5’-aaccctcagagccaccccta-3’ | 5’-gtgcatacaaagcaaactgc-3’ | 2520-2805 | 55 | 27 | 286 |
| MMP-3 | 5’-agctctgaaagtctgggaaggt-3’ | 5’-gtgctgacaggatcaaagg-3’ | 473-972 | 55 | 34 | 499 |
| MMP-7 | 5’-gtttagaagccaaactcaagg-3’ | 5’-ctttgacactaatcgatccac-3’ | 208-440 | 55 | 40 | 232 |
| MMP-9 | 5’-tgggctacgtgacctatgac-3’ | 5’-caaaggtgagaagagagggc-3’ | 2100-2290 | 60 | 33 | 190 |
| MMP-12 | 5’-gtggaatcctagcccatgctt-3’ | 5’-aaccagggtccatcatctgtc-3’ | 578-1285 | 62 | 30 | 707 |
| MMP-13 | 5’-tgccattaccagtctccgaga-3’ | 5’-ggcatgacgcgaacaatacggt-3’ | 901-1415 | 55 | 40 | 514 |
| MT1-MMP | 5’-gcagccaggttaaagctaac-3’ | 5’-cctcactgagatggtgagca-3’ | 563-768 | 50 | 37 | 205 |
| MT2-MMP | 5’-acagaaggaggccgacatcat-3’ | 5’-aagagccagtagcgtcccttt-3’ | 1546-2295 | 60 | 35 | 750 |
| MT3-MMP | 5’-tgcggtgtacctgaccagaca-3’ | 5’-gccaaaaccactggtccttgaa-3’ | 799-1381 | 55 | 37 | 799 |
| OPN | 5’-ccacagtagacacatatgatgg-3’ | 5’-cagggagtttccatgaagccac-3’ | 536-1193 | 58 | 39 | 679 |
| PAI-1 | 5’-aggaccgcaacgtggttttctc-3’ | 5’-agtgctgccgtctgatttgtg-3’ | 104-609 | 59 | 34 | 505 |
| TGM2 | 5’-ggtcaactgcaacgatgacc -3’ | 5’-tcggcccacgctcttagtgc-3’ | 781-1399 | 53 | 33 | 618 |
| TIMP-1 | 5’-aattccgacctcgtcatcag-3’ | 5’-gtttgcaggggatggataaa-3’ | 301-676 | 55.5 | 25 | 375 |
| TIMP-2 | 5’-ctggacgttggaggaaagaa-3’ | 5’-gtcgagaaactcctgcttgg-3’ | 606-950 | 55.5 | 28 | 344 |
| TIMP-3 | 5’-acatttaaagaaaggtctat-3’ | 5’-ccaggacgccttctgcaact-3’ | 354-1021 | 55.5 | 28 | 667 |
| TIMP-4 | 5’-cagaccctgctgacatgaa-3’ | 5’-ttgaagggatgtgatggtca-3’ | 492-1012 | 56 | 40 | 521 |
| tPA | 5’-cccagatcgagactcaaagc-3’ | 5’-atgttctgcccaagatcacc-3’ | 641-1224 | 52 | 30 | 583 |
| αB-Cry | 5’-ttgttcggagagcacctgtt-3’ | 5’-gccagagacctgtttccttg-3’ | 104-487 | 58 | 30 | 384 |
